# Modified scalp block and total intravenous anesthesia for frontal craniotomy in a dog with a meningioma: a technical case report

**DOI:** 10.1007/s11259-026-11502-y

**Published:** 2026-09-09

**Authors:** Pollyana Freisleben, Luiza Tonietto Mangini, Jean Carlos Gasparotto, João Felipe da Silva Mielke, Julya Nathalya Felix Chaves, Amanda Azevedo Dal Pozzolo, Amanda Miwa Takamori Sekita, Diego Vilibaldo Beckmann, Alexandre Mazzanti

**Affiliations:** 1 Veterinary Neurology and Neurosurgery Service/Graduate Program in Veterinary Medicine, University Veterinary Hospital, Universidade Federal de Santa Maria, Santa Maria, 97105900 RS Brazil; 2 Department of Small Animal Clinical, Veterinary Neurology and Neurosurgery Service, Universidade Federal de Santa Maria, Av. Roraima 1000, prédio 97, Santa Maria, CEP 97105900 RS Brasil

**Keywords:** Canine, Craniotomy, Meningioma, Neuroanesthesia, Scalp block, Total intravenous anesthesia

## Abstract

Intracranial meningiomas are among the most common primary central nervous system tumors in dogs, and surgical resection is associated with prolonged survival for rostrotentorial lesions. This report describes the anesthetic and surgical management of a dog undergoing frontal craniotomy using a modified scalp block technique. A 9-year-old male Shih Tzu with seizures and forebrain signs was diagnosed with a rostrotentorial intracranial mass compatible with meningioma on computed tomography. Frontal craniotomy was performed under total intravenous anesthesia with propofol target-controlled infusion combined with bilateral frontal and modified zygomaticotemporal scalp block using bupivacaine. The modified technique involved caudal needle insertion along the zygomatic arch with dorsomedial redirection toward the orbital ligament and fractionated anesthetic deposition. Cardiovascular parameters remained stable during the four major nociceptive events analyzed—skin incision, muscle dissection, craniotomy, and durotomy—with no clinically relevant increases beyond approximately 20% of stabilized post-induction baseline values; no rescue analgesia or adjustments in target propofol plasma concentration were required intraoperatively. During cranioplasty, the patient developed hemodynamic instability associated with blood loss, which improved after transfusion therapy. Postoperative recovery was satisfactory, with neurological improvement and no immediate complications. The association of total intravenous anesthesia and modified scalp block may represent a feasible neuroanesthetic strategy for canine craniotomy, contributing to nociceptive control and cardiovascular stability. Further studies are needed to validate the technique and its analgesic efficacy.

**Supplementary Information:**

The online version contains supplementary material available at https://doi.org/10.1007/s11259-026-11502-y.

## Background

Intracranial neoplasms are an important cause of neurological disease in dogs, particularly in geriatric patients, with meningiomas representing the most frequently diagnosed primary brain tumors (Sturges et al. 2008). Seizures are the most common clinical manifestation in affected dogs, and surgical resection remains the treatment associated with the longest median survival times for rostrotentorial meningiomas (Suñol et al. 2017).

Craniotomy is associated with intense nociceptive stimulation arising from skin incision, muscle dissection, periosteal traction, and cranial osteotomy. These stimuli may elicit marked sympathetic responses, potentially resulting in hemodynamic instability and adverse effects on cerebral physiology. Consequently, anesthetic protocols for intracranial surgery should aim to provide effective analgesia while preserving cardiovascular and intracranial homeostasis. In this context, propofol-based total intravenous anesthesia (TIVA) has gained increasing acceptance because of its favorable effects on cerebral metabolism, cerebral blood flow, and intracranial dynamics, as originally demonstrated by Artru et al. (1992), particularly when combined with locoregional techniques capable of reducing intraoperative nociceptive input and anesthetic requirements (Lee et al. 2006).

In human neuroanesthesia, scalp block is widely used as an adjunctive analgesic technique because it attenuates perioperative nociceptive transmission and contributes to improved cardiovascular stability during craniotomy. The sensory innervation of the canine frontal scalp is primarily provided by branches of the trigeminal nerve, particularly the frontal and zygomaticotemporal nerves, which are directly involved in the transmission of nociceptive stimuli generated during transfrontal surgical approaches. Despite its widespread use in human neurosurgery, reports describing scalp block in dogs remain scarce. Notably, Kushnir et al. (2018) described a landmark-guided regional anesthesia technique targeting the dorsal cranium in dogs; however, standardized scalp block techniques specifically designed for frontal craniotomy have not been established. Furthermore, anatomical variability and the limitations inherent to landmark-guided nerve blocks may affect the accuracy and reliability of local anesthetic deposition. Therefore, technical modifications aimed at improving anesthetic distribution and sensory coverage may represent valuable adjuncts to multimodal neuroanesthetic protocols in veterinary patients undergoing frontal craniotomy.

Accordingly, this report describes the anesthetic and surgical management of a dog undergoing frontal craniotomy for resection of a rostrotentorial meningioma, with particular emphasis on a modified zygomaticotemporal nerve block technique incorporated into a scalp block protocol as a strategy to enhance perioperative analgesia and maintain physiological stability throughout the procedure.

## Case presentation

**Fig. 1:**
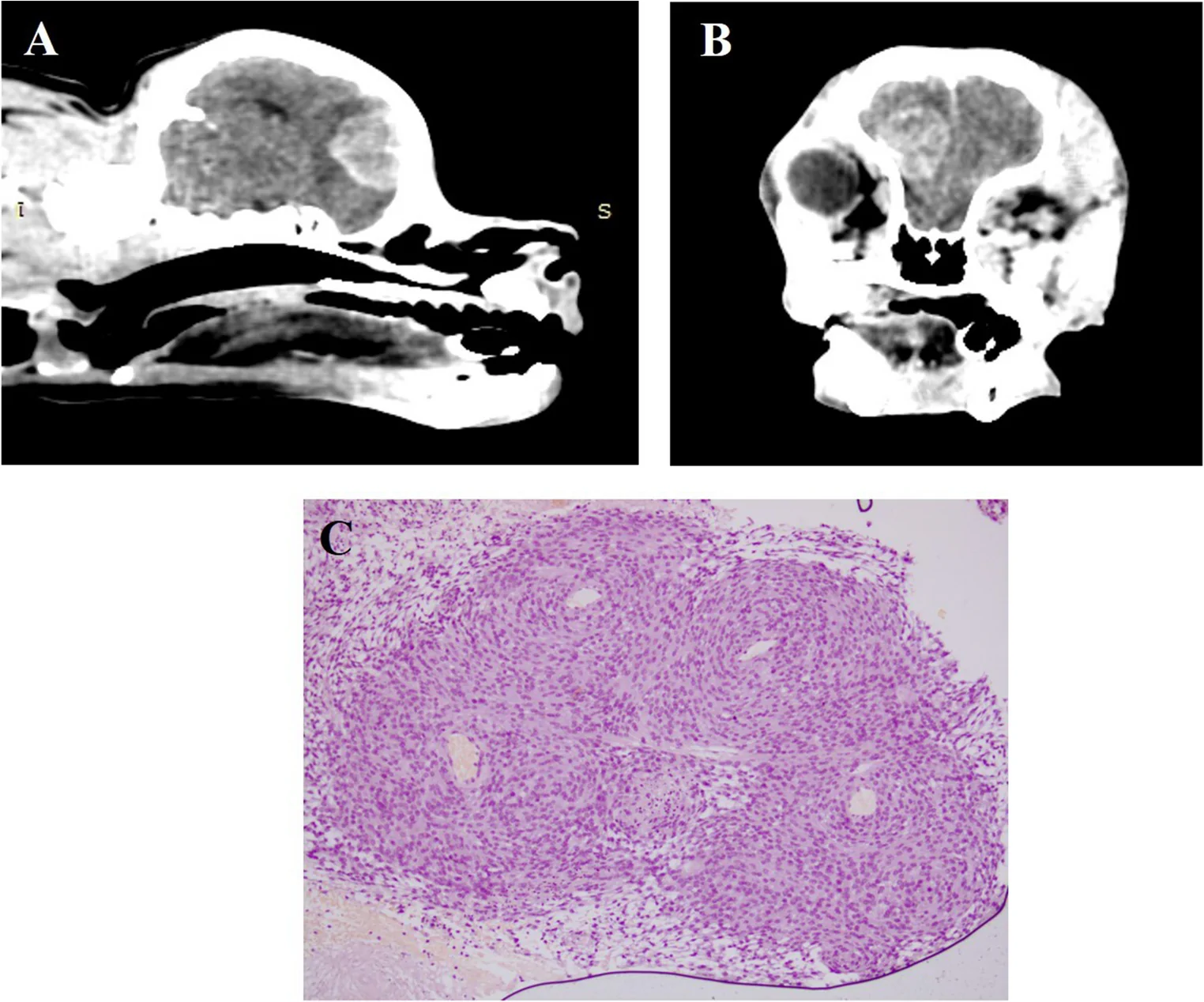
Computed tomography of the brain. Oval-shaped intracranial neoplasm, with regular contours and well-defined margins in the frontal region. Presents soft tissue density, with intravenous contrast enhancement, located in the left frontal lobe, displacing the midline to the right and partially collapsing the left lateral ventricle. Transverse plane **(A)** and sagittal plane **(B)**. Histopathological appearance of a grade I transitional meningioma surgically excised from the canine brain. The neoplasm is composed of well-differentiated meningothelial cells arranged in characteristic concentric whorls and interlacing bundles, supported by a delicate fibrovascular stroma. Neoplastic cells exhibit mild anisocytosis and anisokaryosis, indistinct cell borders, and low mitotic activity, consistent with a low-grade meningioma **(C)**. Hematoxylin and eosin stain (H&E), original magnification 20x

A 9-year-old neutered male Shih Tzu weighing 6.7 kg was referred to the Veterinary Neurology and Neurosurgery Service of the Federal University of Santa Maria with a history of seizures, ambulatory paraparesis, compulsive pacing, circling to the right, and head pressing against walls and obstacles. Computed tomography revealed an oval-shaped intracranial mass with regular contours and well-defined margins located in the frontal region (Fig. 1A and B). Prior to surgery, treatment with phenobarbital and prednisolone was instituted to control seizures and manage secondary effects associated with the intracranial mass (Dewey and da Costa 2016). Following partial improvement of the neurological signs with supportive treatment, the owner elected surgical resection of the lesion.

During preoperative evaluation, the patient exhibited persistent systolic hypertension (240 mmHg), confirmed by repeated measurements. Considering the presence of an intracranial space-occupying lesion, previous corticosteroid administration, and situational stress associated with the hospital environment, the hypertension was considered potentially influenced by these factors. No evidence of target organ damage was identified during the preoperative evaluation. Therefore, no specific antihypertensive treatment was instituted before anesthesia. The dog also presented a grade II/VI mitral murmur without clinically relevant echocardiographic structural abnormalities and sinus arrhythmia. Preoperative laboratory evaluation revealed mild leukocytosis characterized by mature neutrophilia, mild elevation of ALT activity, marked elevation of alkaline phosphatase activity, considered consistent with previous corticosteroid administration, and a slight increase in serum albumin concentration. No clinically relevant abnormalities in renal function or coagulation were identified, and total protein concentration and platelet count were within the respective reference intervals (Tables 1 and 2). The patient was classified as ASA Physical Status III according to the American Society of Anesthesiologists classification system (Portier and Ida 2018).

**Table 1:** Preoperative hematological and coagulation findings

Parameter	Result	Reference interval*
Erythrocytes (×10⁶/µL)	6.08	4,8–9.3
Hemoglobin (g/dL)	15.1	12.0–18.0
Packed cell volume (%)	44.0	36–60
MCV (fL)	72.3	58-79
MCHC (g/dL)	34.3	33-38
Platelets (×10³/µL)	359	200–500
Total plasma protein (g/dL)	7.0	6.0–8.5
Leukocytes (×10³/µL)	18,200	4-15.5
Band neutrophils (×10³/µL)	182	0–0.3
Segmented neutrophils (×10³/µL)	15,843	2-10,5
Lymphocytes (×10³/µL)	1,274	1-4.5
Monocytes (×10³/µL)	728	0–0.9
Eosinophils (×10³/µL)	182	0.1-1.2
Thrombin time (seconds)	5.28	6-12
Activated partial thromboplastin time (seconds)	13.3	10-25

^*^﻿Thrall MA, Weiser G, Allison LW, Campbell TW (2012)

**Table 2:** Preoperative serum biochemical profile

Parameter	Result	Reference interval*
Albumin (g/dL)	3.6	2.7-4.4
Total protein (g/dL)	6.4	5.0–7.4
ALT (U/L)	111.1	12–128
Alkaline phosphatase (U/L)	2838	5–131
Creatinine (mg/dL)	0.70	0.5–1.6
Urea (mg/dL)	39.7	06–25

^*^Thrall MA, Weiser G, Allison LW, Campbell TW (2012)

Dexmedetomidine (3 µg/kg IM) was administered as premedication. After peripheral venous catheterization, anesthesia was induced with midazolam (0.2 mg/kg IV) and propofol delivered using a target-controlled infusion system (TCI; SDA Med^®^, Brazil) with plasma concentration control (Cp). The pharmacokinetic model incorporated into the TCI system corresponded to the model available in the manufacturer’s proprietary software. The initial target plasma concentration was adjusted according to the patient’s clinical response and maintained between 5 and 7 µg/mL throughout the procedure, with subsequent adjustments based on cardiovascular variables, anesthetic depth, and surgical stimulation. Following anesthetic induction, arterial blood pressure decreased without specific antihypertensive intervention, with systolic and mean arterial pressures of 123 and 95 mmHg, respectively, after anesthetic stabilization. Lactated Ringer’s solution was administered at 3 mL/kg/h. Neuromuscular blockade was achieved with atracurium (0.2 mg/kg IV bolus followed by a continuous infusion of 0.3 mg/kg/h IV). No opioids were administered during anesthesia or surgery.

**Fig. 2:**
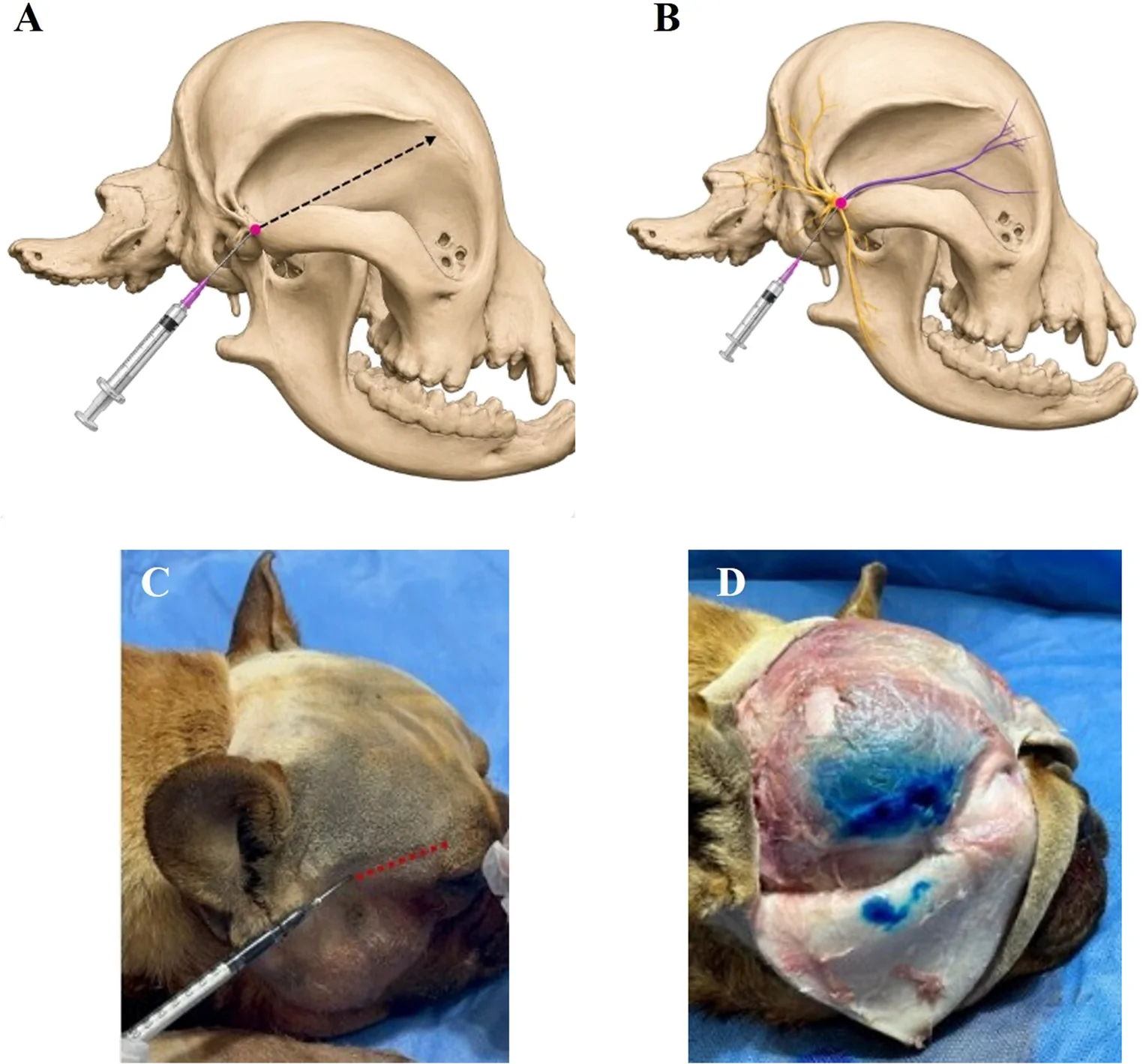
Three-dimensional skull reconstruction illustrating the modified zygomaticotemporal nerve block performed in a dog undergoing unilateral transfrontal craniotomy. **(A)** Needle insertion point and trajectory. **(B)** Anatomical course of the zygomatic nerve and its zygomaticotemporal and zygomaticofacial branches. **(C)** Cadaveric demonstration of the modified zygomaticotemporal nerve block. Needle positioned at the caudal portion of the zygomatic arch and directed dorsomedially toward the orbital ligament; the dashed line indicates the estimated needle path and direction of local anesthetic deposition. **(D)** Anatomical dissection after dye injection, showing distribution of the solution within the periorbital region and along the presumed anatomical course of the zygomaticotemporal nerve

For intraoperative analgesia, bilateral frontal and zygomaticotemporal nerve blocks were performed using 0.5% bupivacaine (0.08 mL/kg per injection site). The total volume of 0.5% bupivacaine administered across the four injection sites (two frontal and two zygomaticotemporal) was 2.14 mL, corresponding to a total dose of 1.6 mg/kg. This dose was calculated to remain within established safety limits for the species. For the zygomaticotemporal nerve block, a modification of the technique originally described by Kushnir et al. (2018) was employed. The modified technique was developed based on preliminary anatomical observations in three canine cadavers (Fig. 2). The original landmark-guided technique described by Kushnir et al. (2018), in which the needle is inserted caudal to the orbital ligament and advanced ventrally, rostrally, and medially toward the nasal planum with local anesthetic deposited at the final position and during needle withdrawal, was initially evaluated in two cadavers (four sides) by contrast injection followed by anatomical dissection. However, contrast distribution was not observed along the presumed anatomical course of the zygomaticotemporal nerve in any of the four evaluated sides. Based on these findings, the approach was modified and subsequently evaluated in three canine cadavers (six sides) using the same methodology. Contrast distribution along the presumed anatomical course of the zygomaticotemporal nerve was observed in all six evaluated sides (Fig. 2C–D). Following palpation of the zygomatic arch, the needle was inserted caudal to the arch and advanced obliquely in a dorsomedial direction toward the orbital ligament. The local anesthetic was deposited incrementally along the needle tract, with an initial deeper injection followed by additional deposition during gradual withdrawal, aiming to enhance local anesthetic distribution along the presumed anatomical course of the zygomaticotemporal nerve (Figs. 2A–D). The total bupivacaine volume administered was calculated to remain within established safety limits for the species and corresponded to the sum of all bilateral frontal and zygomaticotemporal injection sites. The block was performed before skin incision to provide pre-emptive analgesia and minimize transmission of nociceptive stimuli originating from the scalp and superficial cranial structures.

For the assessment of intraoperative physiological responses, baseline cardiovascular values were defined as those recorded after anesthetic induction, following hemodynamic stabilization and immediately before the onset of surgical stimulation. Consequently, intraoperative changes were interpreted relative to these stabilized post-induction values rather than to pre-anesthetic physiological measurements.

Anesthetic monitoring included electrocardiography, pulse oximetry, capnography, body temperature, and invasive arterial blood pressure measurements recorded every five minutes (Suplementar Materials). Mechanical ventilation was adjusted to maintain normocapnia, resulting in a mean end-tidal CO₂ of 32 mmHg and oxygen saturation of 100% throughout the procedure. Cardiovascular variables remained stable during surgery, with a mean arterial pressure of 90 ± 8 mmHg and a mean heart rate of 78 ± 27 beats/min. During major nociceptive surgical events, including skin incision, muscle dissection, craniotomy, and durotomy, no clinically relevant cardiovascular changes suggestive of a marked sympathetic response were observed. Heart rate and mean arterial pressure remained generally stable, with variations of approximately 20% or less from the stabilized post-induction baseline values during these predefined surgical events (Table 3).

**Table 3:** Intraoperative physiological variables recorded during anesthetic management of a dog undergoing transfrontal craniotomy and resection of a grade I transitional meningioma. Heart rate (HR), respiratory rate (f), peripheral oxygen saturation (SpO₂), end-tidal carbon dioxide (ETCO₂), systolic arterial pressure (SAP), mean arterial pressure (MAP), diastolic arterial pressure (DAP), and body temperature (Temp) were monitored throughout the surgical procedure. Values are presented as single measurements or as median (range) during prolonged surgical stages

Surgical Stage	Time(min)	HR (bpm/min)	f (br/min)	SpO₂ (%)	ETCO₂ (mmHg)	SAP(mmHg)	MAP (mmHg)	DAP(mmHg)	Temp (°C)
Baseline	0	98	20	99	38	123	95	80	37,8
Skin incision	5	48	20	100	31	129	93	76	37,4
Muscle dissection	10	49	20	100	31	128	90	78	37,4
Craniotomy	15-35	58(50–65)	20	100	32(27–35)	123(121–128)	89(87–92)	72(70–75)	37,4(37,3–37,5)
Durotomy	40	54	20	100	31	121	87	71	37,2
Tumor resection	45-90	57(52–111)	20	100	32(31–33)	130(127–149)	93(87–107)	74(70–91)	36,8(36,8–37,2)
Cranioplasty	95-120	111(85–121)	20	100	32(32–33)	132(118–139)	82(80–97)	74(70–81)	36,7(36,7–36,8)
Skin suture	125-140	99(75–124)	20	100	32(32–41)	131(125–137)	78(76–84)	83 (72–86)	36,7(36,7–36,7)

A left unilateral frontal craniotomy was performed as previously described by Uriarte and Cappello (2017), using a pneumatic drill under continuous saline irrigation, a 2-mm Kerrison rongeur, and a bone rongeur. Dural incision was subsequently performed, followed by en bloc tumor resection. At the end of the procedure, cranial reconstruction was achieved using pre-molded polymethylmethacrylate (Akin and Shores 2017). Histopathological examination confirmed the diagnosis of a grade I transitional meningioma (Fig. 1C).

During the cranioplasty stage, the patient developed progressive tachycardia accompanied by a downward trend in arterial blood pressure and marked hyperlactatemia (11.2 mmol/L). Although arterial pressure values did not consistently meet conventional criteria for hypotension, the concurrent cardiovascular changes and marked increase in blood lactate concentration raised clinical concern for impaired systemic perfusion and possible intraoperative blood loss.

Initial treatment consisted of crystalloid volume expansion (10 mL/kg over 15 min). Because the hemodynamic alterations persisted and intraoperative blood loss was clinically suspected, whole-blood transfusion was instituted. Progressive cardiovascular stabilization was observed following transfusion. The volume of blood loss was not objectively quantified, and no intraoperative PCV, total solids, or hemoglobin measurements were available at the time of the event.

In the immediate postoperative period, the patient remained hospitalized for 48 h for neurological monitoring and postoperative analgesic management. No rescue analgesia was required based on clinical assessment during hospitalization. Upon discharge, a multimodal analgesic protocol was prescribed, consisting of dipyrone (500 mg/mL; 25 mg/kg PO every 8 h for 5 days) and tramadol (100 mg/mL; 10 mg/kg PO every 8 h for 5 days). Anti-inflammatory therapy consisted of prednisolone (5 mg tablets; 0.7 mg/kg PO every 24 h). Anticonvulsant treatment included phenobarbital (40 mg/mL; 7.1 mg/kg PO every 12 h) and levetiracetam (100 mg/mL; 20 mg/kg PO every 8 h), both maintained throughout the postoperative period. Postoperative antimicrobial therapy consisted of cephalexin (250 mg/5 mL; 30 mg/kg PO every 8 h for 7 days).

At the 4-day postoperative re-evaluation, the dog exhibited a normal neurological examination, and the owner reported a reduction in seizure frequency compared with the preoperative period. Long-term anticonvulsant therapy with phenobarbital (40 mg/mL; 7.0 mg/kg PO every 12 h) was continued. No anesthetic- or surgery-related complications were identified during hospitalization or at the early postoperative follow-up examination.

## Discussion

Meningiomas are among the most frequently diagnosed primary central nervous system tumors in dogs and are typically identified in middle-aged to geriatric patients (Schöniger et al. 2013). Advances in diagnostic imaging and neurosurgical techniques have expanded the application of intracranial surgery in veterinary medicine (Surgess and Dickson 2026). In dogs with rostrotentorial meningiomas, surgical resection remains the treatment associated with the longest survival times, although factors such as midline shift, ventricular compression, and tumor location may adversely affect prognosis (Suñol et al. 2017). Consequently, anesthetic management represents a critical component of perioperative care, aiming to preserve cerebral perfusion while maintaining adequate hemodynamic, ventilatory, and intracranial homeostasis throughout surgery (Raisis and Bradbrook 2026).

In the present case, dexmedetomidine was selected as premedication because of its analgesic, sympatholytic, and anti-inflammatory properties, which may be advantageous in neurosurgical patients by attenuating stress responses and reducing anesthetic requirements (Abdelwahab et al. 2026; Feng et al. 2021). Total intravenous anesthesia (TIVA) with propofol was chosen because of its favorable effects on cerebral physiology, including reductions in cerebral blood flow, intracranial pressure, cerebral metabolic rate, and electroencephalographic activity (Artru et al. 1992). Compared with inhalational anesthesia, propofol-based TIVA has been associated with improved control of cerebral hemodynamics and preservation of cerebrovascular responsiveness to carbon dioxide (Artru et al. 1992; Bini et al. 2023). For these reasons, the combination of TIVA and locoregional anesthesia has become an increasingly attractive component of multimodal neuroanesthetic protocols in both human and veterinary medicine (Moharari et al. 2024).

Craniotomy is associated with substantial nociceptive stimulation resulting from skin incision, muscle dissection, periosteal traction, and cranial osteotomy. These stimuli may trigger sympathetic activation, leading to hypertension, tachycardia, and secondary increases in intracranial pressure. Therefore, effective perioperative analgesia is particularly important in neurosurgical patients. In human medicine, scalp block is widely used as an adjunctive analgesic technique because it attenuates nociceptive input originating from the scalp and periosteum, which are recognized as major sources of pain during craniotomy. Previous studies have demonstrated reduced cardiovascular responses to surgical stimulation and decreased perioperative opioid consumption in patients receiving scalp blocks during elective craniotomy, supporting their role in opioid-sparing anesthetic strategies (Moharari et al. 2024; Carella et al. 2021).

In the present case, cardiovascular variables remained stable during the principal nociceptive stages of surgery, including skin incision, muscle dissection, craniotomy, and durotomy (Table 3). Furthermore, no rescue analgesia or increases in target propofol plasma concentration were required during periods of greater surgical stimulation. Although the individual contribution of the scalp block cannot be isolated from the effects of dexmedetomidine administration and propofol-based TIVA, these observations are consistent with adequate perioperative nociceptive modulation achieved through the multimodal anesthetic approach employed.

In addition to its potential anesthetic- and opioid-sparing effects, the scalp block may have contributed to pre-emptive analgesia by reducing nociceptive input before surgical stimulation occurred. In human neurosurgical patients, pre-emptive analgesic techniques have been associated with reduced sympathetic activation, lower perioperative analgesic requirements, and improved physiological stability during craniotomy procedures (Carella et al. 2021). Although objective nociception monitoring was not available in the present case, the absence of clinically detectable cardiovascular responses during major surgical stimuli is consistent with a potential contribution of the scalp block to perioperative nociceptive modulation.

The analgesic protocol was based on blockade of the frontal and zygomaticotemporal branches of the trigeminal nerve (Giuliano and Walsh 2013). The classical zygomaticotemporal nerve block described in canine cadaveric studies involves needle insertion perpendicular to the zygomatic arch, caudal to the orbital ligament, followed by ventral, rostral, and medial advancement of the needle (Kushnir et al. 2018). In contrast, the modified technique described herein employed a more caudal insertion point, dorsomedial needle redirection, and incremental local anesthetic administration during both needle advancement and withdrawal.

This modification was derived from previous anatomical observations in canine cadavers, in which the original technique did not consistently produce satisfactory dye distribution along the presumed course of the zygomaticotemporal nerve. The rationale for the modified approach is related to the anatomical variability of this nerve and the inherent limitations of landmark-guided techniques. Similar adaptations have been described in human medicine with the objective of improving local anesthetic spread and increasing block reliability during cranial procedures (Sato et al. 2025). Therefore, the modified approach was designed to enhance anesthetic distribution throughout the sensory territory involved in the transfrontal surgical approach. Given the potential for anatomical variation and early branching of the zygomaticotemporal nerve, techniques that promote broader anesthetic dispersion may represent useful alternatives for scalp sensory blockade during frontal craniotomies.

During the cranioplasty stage, the patient developed hypotension, tachycardia, and marked hyperlactatemia. Although intraoperative hemorrhage associated with systemic hypoperfusion was considered the most likely explanation, the contribution of other physiological factors cannot be completely excluded. Nevertheless, progressive hemodynamic improvement following whole-blood transfusion supports the hypothesis that circulatory compromise secondary to blood loss played a major role in the observed abnormalities.

Several limitations should be considered when interpreting the findings of this report. Direct intracranial pressure monitoring was not performed, preventing objective assessment of the effects of the anesthetic protocol on intracranial dynamics. Likewise, objective intraoperative nociception monitoring tools (e.g., the Analgesia Nociception Index, pupillometry, or the nociceptive flexion reflex) were unavailable, limiting direct evaluation of nociceptive responses during surgery. Neuromuscular transmission monitoring was also not performed during continuous atracurium administration. Furthermore, the individual analgesic contributions of the frontal and zygomaticotemporal nerve blocks could not be determined because of their close anatomical relationship. The absence of validated postoperative pain scoring systems and a control group further limits the objective assessment of analgesic efficacy and precludes definitive conclusions regarding the superiority of the proposed technique over conventional protocols.

Despite these limitations, the modified zygomaticotemporal nerve block was technically feasible and was successfully incorporated into a multimodal neuroanesthetic protocol for frontal craniotomy. The favorable perioperative course observed in this patient suggests that the technique may represent a useful adjunctive strategy for perioperative analgesia in canine neurosurgery. However, anatomical, cadaveric, and prospective clinical studies are required to validate the technique, characterize local anesthetic distribution, and objectively assess its reproducibility, analgesic efficacy, and potential anesthetic-sparing effects.

## Conclusion

The combination of propofol-based total intravenous anesthesia and a modified scalp block was feasible during frontal craniotomy in a dog with a rostrotentorial meningioma and was associated with a clinically satisfactory perioperative course. Cardiovascular variables remained generally stable during the major nociceptive surgical events, and no rescue analgesia or intraoperative opioids were required. However, given the multimodal nature of the anesthetic protocol, the specific contribution of the scalp block to these findings cannot be determined.

The modified zygomaticotemporal nerve block described herein was technically feasible in this clinical setting and may represent a useful adjunct to multimodal neuroanesthetic protocols for canine frontal craniotomy. These findings should be considered hypothesis-generating rather than evidence of analgesic efficacy or hemodynamic benefit. Further anatomical, cadaveric, and prospective clinical studies are required to validate the technique, characterize local anesthetic distribution, and objectively assess its analgesic efficacy, reproducibility, and potential anesthetic-sparing effects.

## Supplementary Information

Below is the link to the electronic supplementary material.

Supplementary Material 1 Hemodynamic, respiratory, and anesthetic variables recorded during the neurosurgical procedure. Heart rate (HR), respiratory rate (f), peripheral oxygen saturation (SpO₂), end-tidal carbon dioxide (ETCO₂), systolic arterial pressure (SAP), mean arterial pressure (MAP), diastolic arterial pressure (DAP), and body temperature (Temp). (DOCX 18 KB)

## Data Availability

No datasets were generated or analysed during the current study.
