# Supplementary material for: Modified scalp block and total intravenous anesthesia for frontal craniotomy in a dog with a meningioma: a technical case report
Source: Vet Res Commun. 2026 Sep 9;50(6):557. doi: 10.1007/s11259-026-11502-y (PMC13558315; doi:10.1007/s11259-026-11502-y)
Supplement: Supplementary file 1 [file 11259_2026_11502_MOESM1_ESM.docx]

| **Time**  **(min)** | **HR (bpm/min)** | ***f***  **(br/min)** | **SpO₂ (%)** | **ETCO₂ (mmHg)** | **SAP**  **(mmHg)** | **MAP (mmHg)** | **DAP**  **(mmHg)** | **Temp (°C)** |
| --- | --- | --- | --- | --- | --- | --- | --- | --- |
| 0 | 98 | 20 | 99 | 38 | 123 | 95 | 80 | 37,8 |
| 5 | 48 | 20 | 100 | 31 | 129 | 93 | 76 | 37,4 |
| 10 | 49 | 20 | 100 | 31 | 128 | 90 | 78 | 37,4 |
| 15 | 50 | 20 | 100 | 32 | 128 | 92 | 75 | 37,5 |
| 20 | 61 | 20 | 100 | 35 | 123 | 90 | 71 | 37,4 |
| 25 | 65 | 20 | 100 | 33 | 123 | 89 | 70 | 37,4 |
| 30 | 56 | 20 | 100 | 28 | 122 | 88 | 72 | 37,3 |
| 35 | 58 | 20 | 100 | 27 | 121 | 87 | 75 | 37,3 |
| 40 | 54 | 20 | 100 | 31 | 121 | 87 | 71 | 37,2 |
| 45 | 52 | 20 | 100 | 31 | 130 | 90 | 74 | 37,2 |
| 50 | 54 | 20 | 100 | 32 | 130 | 90 | 72 | 37,1 |
| 55 | 52 | 20 | 100 | 32 | 130 | 90 | 70 | 36,8 |
| 60 | 54 | 20 | 100 | 32 | 129 | 89 | 73 | 36,8 |
| 65 | 55 | 20 | 100 | 32 | 127 | 87 | 72 | 36,8 |
| 70 | 57 | 20 | 100 | 32 | 128 | 97 | 74 | 36,8 |
| 75 | 62 | 20 | 100 | 33 | 137 | 101 | 81 | 36,8 |
| 80 | 94 | 20 | 100 | 33 | 137 | 99 | 85 | 36,8 |
| 85 | 111 | 20 | 100 | 32 | 149 | 107 | 88 | 36,8 |
| 90 | 97 | 20 | 100 | 32 | 147 | 104 | 91 | 36,8 |
| 95 | 85 | 20 | 100 | 32 | 139 | 97 | 81 | 36,8 |
| 100 | 106 | 20 | 100 | 32 | 138 | 92 | 77 | 36,7 |
| 105 | 108 | 20 | 100 | 32 | 136 | 83 | 76 | 36,7 |
| 110 | 118 | 20 | 100 | 33 | 128 | 80 | 73 | 36,7 |
| 115 | 114 | 20 | 100 | 32 | 118 | 83 | 70 | 36,7 |
| 120 | 121 | 20 | 100 | 32 | 126 | 82 | 73 | 36,7 |
| 125 | 115 | 20 | 100 | 32 | 125 | 84 | 72 | 36,7 |
| 130 | 124 | 20 | 100 | 32 | 126 | 76 | 84 | 36,7 |
| 135 | 83 | 20 | 100 | 40 | 137 | 78 | 86 | 36,7 |
| 140 | 75 | 20 | 100 | 41 | 137 | 78 | 82 | 36,7 |
